# Trends and inequities in fall- and musculoskeletal disorder–related mortality among U.S. adults aged 65 years and older, 1999–2023

**DOI:** 10.3389/fpubh.2026.1804068

**Published:** 2026-04-22

**Authors:** LuLu Zhang, Yan Wang, Dong Wang, XueZhi Liu, YanXin Li, JianXiong Ma

**Affiliations:** 1 School of Integrated Traditional Chinese and Western Medicine, Tianjin University of Traditional Chinese Medicine, Tianjin, China; 2 Tianjin Hospital, Tianjin University, Tianjin, China; 3 Tianjin Orthopedic Institute, Tianjin, China

**Keywords:** CDC WONDER, older adults, falls, mortality trends, musculoskeletal disorders

## Abstract

**Background:**

Musculoskeletal disorders (MSDs) and falls increasingly threaten U.S. older adults. Contemporary trend analyses are essential for targeted prevention.

**Objective:**

To assess national and subgroup trends in aggregate mortality attributable to MSDs or falls among U.S. adults aged ≥65 years (1999–2023).

**Methods:**

Analyzing CDC WONDER data, deaths with an underlying cause of MSDs or falls were aggregated. We calculated age-adjusted mortality rates (AAMRs) per 100,000 and estimated average annual percent changes (AAPCs) using joinpoint regression, stratified by year, sex, age group, race/ethnicity, state, Census region, and urban–rural classification.

**Results:**

From 1999 to 2023, the composite AAMR rose from 51.73 to 98.30 (AAPC = 2.87%). Disaggregated analysis revealed a historic divergence followed by a recent synergistic surge: post-2017, both MSD- and fall-specific mortalities accelerated concurrently (APCs 4.51% and 3.19%). In 2023, the AAMR and AAPC were 111.65 and 2.94% for men, and 88.13 and 2.56% for women, respectively. Non-Hispanic White adults recorded the highest AAMR (110.94). Geographically, the South (AAPC = 3.23%) and non-metropolitan counties (2020 AAMR: 92.17 vs. 85.82) faced the heaviest burdens. State-level AAPCs peaked in Oklahoma (5.70%) and Vermont (5.30%).

**Conclusions:**

The macroscopic mortality burden of musculoskeletal decline and falls is worsening. The recent concurrent acceleration in both domains indicates a systemic breakdown in geriatric mobility preservation, highlighting an urgent need for integrated public health interventions targeting high-risk demographics and regions.

## 1 Introduction

Musculoskeletal disorders (MSDs) and falls constitute critical public-health challenges for older adults in the United States (1, 2). Driven by a rapidly aging demographic—projected to increase from 56 million adults aged ≥65 years in 2020 to 94.7 million by 2060—these interconnected conditions increasingly threaten the sustainability of social and economic systems (3). Beyond their individual burdens on healthcare expenditures and quality of life, the mutual interplay between MSDs and falls has intensified, underscoring the need for rigorous, integrated surveillance of long-term mortality trends to inform targeted prevention and policy.

From a pathophysiological perspective, MSDs—including osteoporosis, arthritis, and degenerative joint disease—exhibit a bidirectional relationship with fall risk (4). MSDs are intrinsically associated with muscle weakness, imbalanced gait, and reduced joint range of motion, thereby increasing vulnerability to falls (5). Conversely, falls frequently cause traumatic fractures, particularly to the hip and spine, which abruptly accelerate sarcopenia, deformity, and functional decline. This forms a catastrophic “disease–injury–disability” cycle (5). Surveillance data indicate that approximately one in four U.S. adults aged ≥65 years report at least one fall annually (6–8). Following severe fall-related injuries such as hip fractures, less than half of older adults recover to their pre-injury activity levels within a year, and many progress to persistent mobility limitations or institutional care (7, 9, 10). These conjoint clinical outcomes exert sustained pressure on long-term services and impose a growing economic burden on public payers. Combined medical costs of fatal and non-fatal falls among older adults routinely approach or exceed $50 billion annually in the U.S. (11–13). Concurrently, secular shifts in key risk factors have further reshaped this epidemiology. Over the past two decades, the prevalence of obesity has remained high (≈39% among those aged ≥60 years), physical inactivity persists, and polypharmacy has become increasingly common (14–16). Each of these factors exacerbates muscle weakness, compromises bone mineral density, and increases medication-related fall risk, functioning as catalysts that potentially alter mortality trajectories.

Despite broad recognition of this public-health crisis, several critical evidence gaps limit the precision of decision-making. First, systematic assessments of long-term mortality trends remain scarce. Many prior studies focus on single time points or short intervals, leaving the national trajectory over the last quarter-century (1999–2023) insufficiently characterized. Second, subgroup analyses are often incomplete or utilize variable definitions, limiting the identification of persistent demographic and geographic patterns (6–8). Third, high-risk populations—particularly regarding urban–rural disparities and precise racial/ethnic heterogeneity—require validation using standardized national time series to enable optimal resource targeting (6–8). Fourth, and most critically, traditional epidemiological surveillance has analyzed MSDs and falls separately. However, because MSDs and falls share an intensely interconnected pathological cycle and target the exact same vulnerable aging population, they heavily burden the same geriatric, orthopedic, and rehabilitation health-system resources (17–19). Therefore, tracking their aggregate mortality trajectory is conceptually essential. Viewing either condition in isolation risks obscuring the true public health burden due to “diagnostic shifting”—where pharmacological improvements might prevent spontaneous MSD-related deaths, only for the same vulnerable population to subsequently die from fatal falls (20, 21). Because both pathways ultimately converge on the exact same catastrophic endpoint and overwhelm the same geriatric and orthopedic resources, measuring their combined macroscopic burden provides policymakers with a much more accurate gauge of “musculoskeletal and mobility failure” in the aging U.S. population. Accordingly, the primary objective of this study was to assess national and subgroup trends in the aggregate mortality burden attributable to MSDs or falls among U.S. adults aged ≥65 years from 1999 to 2023. To achieve this, we deliberately utilized the Underlying Cause of Death (UCOD) database rather than Multiple Cause of Death (MCOD) records. The UCOD strictly identifies the initiating root cause of the fatal cascade (i.e., dying from an MSD or fall). Because chronic MSDs like osteoarthritis are ubiquitous background conditions in older adults, relying on MCOD would introduce massive co-morbidity bias by capturing patients who died of unrelated acute events (e.g., myocardial infarction) but merely died with an MSD. Furthermore, the mutually exclusive nature of UCOD coding provides a mathematically clean foundation for our composite endpoint, ensuring absolute zero double-counting when aggregating these two interconnected clinical domains (22).

**Figure 1:**
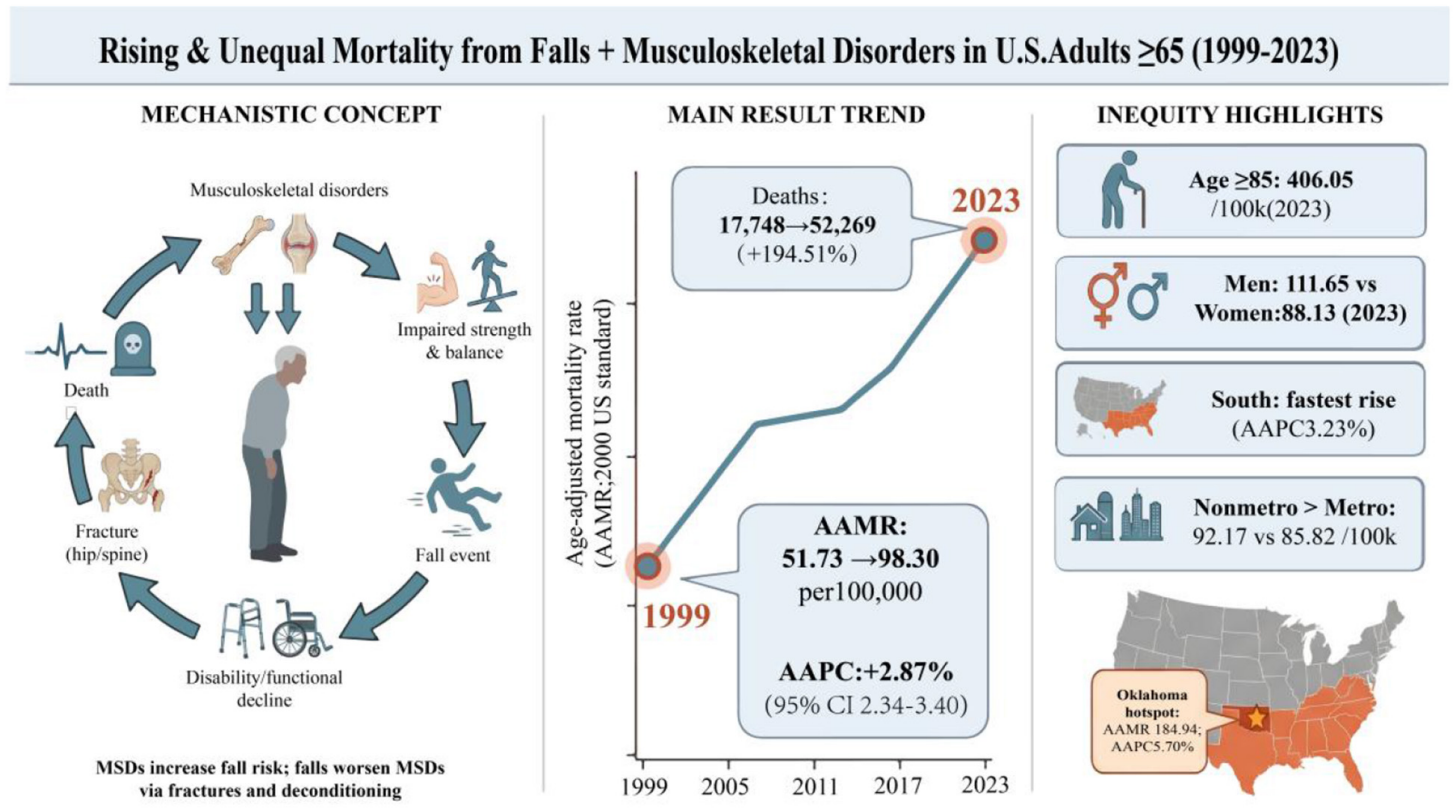
MSD–Fall interplay and public health implications in older adults (≥65 y): framework diagram.

To achieve this, the analysis proceeds in three methodological steps. First, age-adjusted mortality rates (AAMRs) per 100,000 population are computed, and joinpoint regression is utilized to estimate the average annual percent change (AAPC) to identify statistically significant inflection points (23). Furthermore, to ensure transparency and identify the distinct pathophysiological drivers of the composite burden, we pre-specified disaggregated time-series analyses of fall-specific and MSD-specific underlying mortalities. Second, comprehensive stratification is undertaken by sex, age, race/ethnicity, Census region, state, and urban–rural classification to reveal population- and place-based disparities (6–8). Third, we synthesize these findings into a conceptual model (Figure 1) to summarize the bidirectional causality and resulting clinical-economic chain.

## 2 Materials and methods

### 2.1 Study design and data source

A population-based retrospective cohort analysis was conducted using the Centers for Disease Control and Prevention (CDC) Wide-Ranging Online Data for Epidemiologic Research (WONDER) Underlying Cause of Death database (22). This platform aggregates standardized death certificate records from all U.S. jurisdictions and undergoes federal–state quality assurance procedures, including automated logic checks, internal consistency validation, and cross-year harmonization of coding. Data encompass decedents' demographic characteristics and their single, mutually exclusive underlying cause of death, coded using the International Classification of Diseases, Tenth Revision (ICD-10) (24). Per World Health Organization (WHO) coding rules, the underlying cause of death is strictly defined as the single disease or injury that initiated the train of morbid events leading directly to death. All extraction and processing steps were performed according to a pre-specified protocol to maximize reproducibility.

### 2.2 Study period and target population

The observation window spanned January 1, 1999 through December 31, 2023. The analytic population comprised all decedents aged ≥65 years at the time of death, consistent with the National Institutes of Health (NIH) convention for older adults. Records were excluded if (i) essential demographic variables (age, sex, race/ethnicity) were missing; (ii) the decedent was a resident of Alaska. Alaska was excluded *a priori* from the analyses because the annual death counts for our outcome were fewer than 20 for multiple years (specifically, 1999, 2000, 2002, 2003, and 2006) (22). According to the CDC National Center for Health Statistics (NCHS) guidelines, age-adjusted mortality rates based on fewer than 20 deaths are statistically unreliable and are suppressed or flagged accordingly in the WONDER database. Including these years would preclude stable, continuous joinpoint regression modeling at the state level (22). Consequently, all national and subnational geographic summaries refer to the 49 states plus the District of Columbia. All national and subnational summaries therefore refer to the 49 states plus the District of Columbia; where regional estimates are presented, Census region denominators were recalculated to exclude Alaska to preserve internal consistency.

### 2.3 Outcome definitions

The primary outcomes were defined *a priori* based on the International Classification of Diseases, Tenth Revision (ICD-10). Musculoskeletal disorders (MSDs) were identified using Chapter XIII codes M00–M99, encompassing arthropathies (M00–M25), dorsopathies (M40–M54), soft-tissue disorders (M60–M79), osteopathies and chondropathies (M80–M94), and other disorders of the musculoskeletal system (M95–M99). Fall-related mortality was identified using external cause codes W00–W19, capturing fatal falls across various mechanisms and settings (e.g., fall on the same level from slipping, tripping, and stumbling, W01; fall involving a bed, W06). To ensure maximum methodological rigor, mortality cases were extracted strictly based on the Underlying Cause of Death (UCOD). The UCOD is defined by the World Health Organization (WHO) as the specific disease or injury that initiated the train of morbid events leading directly to death. Under the mutually exclusive coding rules enforced by the CDC WONDER system, a single decedent is administratively assigned exactly one UCOD (22). Consequently, there is absolute zero overlap between MSD-coded and fall-coded underlying deaths in our dataset, precluding any possibility of double-counting. Rather than viewing these conditions in isolation, we calculated the aggregate mortality burden as the arithmetic sum of UCOD-designated MSD deaths and UCOD-designated fall deaths. This composite endpoint was deliberately constructed to evaluate the total macroscopic burden of “musculoskeletal vulnerability and mobility failure”—two clinically interconnected pathways that ultimately converge on the exact same downstream systemic collapse (7, 25).

### 2.4 Ethical considerations

CDC WONDER provides publicly available, fully de-identified mortality data with direct identifiers removed. In accordance with institutional policy and federal regulations for research using de-identified public data, the study qualified for exemption from institutional review board oversight. Reporting followed the STROBE guidance and recent calls to strengthen transparency for observational studies through prospective registration and complete reporting checklists (26).

### 2.5 Variable extraction and classification

A standardized extraction plan was implemented to ensure consistent variable definitions:

**Demographic variables**.

Year of death: calendar year (1999–2023).

Sex: male or female, as recorded on the death certificate.

Age group: 65–74, 75–84, and ≥85 years, following widely used gerontologic strata.

**Race/ethnicity**.

Categories followed the 2000 U.S. Office of Management and Budget (OMB) standards and were operationalized in CDC WONDER as Hispanic/Latino, non-Hispanic White, non-Hispanic Black/African American, non-Hispanic American Indian/Alaska Native, and non-Hispanic Asian/Pacific Islander. Race/ethnicity was analyzed as mutually exclusive categories.

**Geography**.

State: 49 states plus the District of Columbia (Alaska excluded as noted).

Census region: Northeast, Midwest, South, and West, using U.S. Census Bureau 2010 definitions but computed for the Alaska-excluded universe.

Urban–Rural classification: The 2013 National Center for Health Statistics (NCHS) Urban–Rural Classification Scheme for Counties was applied. Metropolitan counties were categorized as large central metro, large fringe metro, medium metro, or small metro; nonmetropolitan counties were categorized as micropolitan or noncore.

Urbanization time window. Because age-adjusted rates in CDC WONDER are actively suppressed by the system for recent datasets when county-level locations (including the 2013 urbanization categories) are selected, automated AAMRs were strictly unavailable for the 2021–2023 period (22). Therefore, to ensure robust age-standardization and comparability across strata, urban–rural analyses were rigorously restricted to data from 1999–2020; where applicable, recent national surveillance reports have applied similar database-driven constraints.

Population denominators: Annual population estimates were taken from the U.S. Census Bureau intercensal and postcensal files as disseminated through CDC WONDER. When computing age-specific rates, age-stratum–specific denominators were used (22, 27).

### 2.6 Data quality assurance

A double-extraction, cross-check, and adjudication workflow was used for all data pulls and tabulations. Two analysts independently executed the CDC WONDER queries and reproduced the exported tables; discrepancies were resolved by a third reviewer. Final agreement across all fields was 99.2%. Data cells suppressed by CDC WONDER for confidentiality (small counts) were aggregated to a higher geographic or demographic level to avoid suppression; no back-calculation of suppressed cells was attempted. Data dictionaries, query strings, and codebooks were version-controlled and archived to support auditability.

### 2.7 Mortality rate calculations

Age-adjusted mortality rates (AAMRs). AAMRs were computed per 100,000 population using the direct standardization method with the 2000 U.S. standard population as the reference, consistent with current NCHS technical guidance. AAMRs were calculated for the total population and for strata where age adjustment was supported by CDC WONDER outputs (e.g., by sex, race/ethnicity, state, and Census region, and by urban–rural categories for 1999–2020).

Crude mortality rates for age groups. For analyses stratified by the three age groups (65–74, 75–84, ≥85 years), crude mortality rates (CMRs) per 100,000 were computed as deaths divided by the corresponding age-specific population denominators. Because the CDC WONDER system provides age adjustment primarily for broad, composite populations rather than within narrow age strata, age-group-specific AAMRs could not be methodologically derived (22, 27). Consequently, AAMRs were strictly replaced by crude mortality rates for all age-specific subgroup analyses. And all subsequent trend estimates (APCs and AAPCs) for these strata were computed using these crude rates.

### 2.8 Trend analysis

Temporal trends were characterized using joinpoint regression (Joinpoint Regression Program, National Cancer Institute, version 5.1.0) (28, 29). Annual AAMRs were modeled on the log scale using weighted least squares, with weights proportional to the inverse of the rate variance to account for heteroscedasticity across years and strata. The optimal number of joinpoints (i.e., statistically significant inflection points) was selected using the weighted Bayesian information criterion (WBIC). For each linear segment, the annual percent change (APC) and its 95% confidence interval (CI) were estimated; a CI excluding zero was interpreted as statistically significant. The average annual percent change (AAPC) and 95% CI over the full study period (or over pre-specified subperiods defined by the model) were then derived to summarize the overall trend. Statistical inference relied on Monte Carlo permutation procedures with 10,000 replicates, as implemented in the Joinpoint software. Model diagnostics included inspection of residual plots and assessment of leverage and influence; no evidence of systematic lack-of-fit was observed in the final models.

### 2.9 Stratified analyses

All rates and trend estimates were generated by year and were further stratified by sex, age group, race/ethnicity, state, Census region, and urban–rural category. For urbanization-stratified analyses, all computations were restricted to 1999–2020 as described above. When strata produced unstable estimates due to small numerators, adjacent categories were aggregated to achieve stable rates while preserving the substantive interpretation (e.g., combining micropolitan and noncore counties where necessary); such aggregations are noted in the corresponding tables or figures.

### 2.10 Software and statistical significance

All data management and descriptive analyses were conducted in R version 4.5.1 (R Foundation for Statistical Computing). Trend modeling was performed in the National Cancer Institute Joinpoint Regression Program (version 5.1.0) (29). Two-sided statistical significance was defined as *p* < 0.05. Because the primary goal was surveillance and estimation rather than hypothesis testing, and because APC/AAPC inference used permutation-based CIs, adjustments for multiple comparisons were not applied; effect sizes and CIs are therefore emphasized in interpretation.

### 2.11 Transparency and reproducibility

The full set of CDC WONDER query parameters (cause-of-death code lists, age and race/ethnicity recodes, geographic selections, and group-by fields), variable recoding scripts, and data processing logs were archived and are available on request. All analyses adhered to a pre-specified plan that defined inclusion/exclusion criteria, outcomes, and stratifications prior to data extraction. The final analytic dataset and code enable complete reproduction of the tables and figures presented in Section 3.

## 3 Results

### 3.1 Overall trend

From 1999 to 2023, deaths attributed to musculoskeletal disorders and falls increased from 17,748 to 52,269 (relative change: +194.51%). The composite AAMR rose from 51.73 to 98.30 per 100,000 (AAPC, 1999–2023: 2.87%, 95% CI 2.34–3.40; *p* < 0.001), indicating a statistically significant long-term increase. Full estimates are provided in Table 1.

**Table 1:** Musculoskeletal disorders and Fall-related deaths and AAMR in the United States from 1999 to 2023 and their changing trends.

Characteristic	Deaths			AAMR			
	1999	2023	Percent change (%)	1999 (95% CI)	2023 (95% CI)	AAPC (95% CI)	*P*
Total	17,748	52,269	194.51	51.73 (50.97 to 52.49)	98.30 (97.45 to 99.15)	2.87 (2.34 to 3.40)	< 0.001
Sex							
Female	10,942	27,515	151.46	49.32 (48.39 to 50.24)	88.13 (87.09 to 89.18)	2.56 (2.04 to 3.08)	< 0.001
Male	6,806	24,754	263.71	55.41 (54.07 to 56.75)	111.65 (110.23 to 113.06)	2.94 (2.40 to 3.48)	< 0.001
Census Region							
Northeast	3,213	8,943	178.34	43.27 (41.77 to 44.77)	88.47 (86.63 to 90.31)	3.19 (2.28 to 4.10)	< 0.001
Midwest	5,176	13,236	155.72	61.77 (60.08 to 63.45)	117.65 (115.64 to 119.66)	2.95 (2.32 to 3.58)	< 0.001
South	5,581	19,618	251.51	46.90 (45.67 to 48.13)	98.08 (96.70 to 99.46)	3.23 (2.50 to 3.97)	< 0.001
West	3,778	10,472	177.18	57.22 (55.39 to 59.04)	88.41 (86.71 to 90.11)	1.82 (1.29 to 2.36)	< 0.001
Race							
Hispanic	566	2,763	388.16	40.81 (37.38 to 44.23)	57.92 (55.74 to 60.11)	1.49 (0.25 to 2.75)	0.018
NH Black	958	2,920	204.80	35.94 (33.66 to 38.23)	60.34 (58.10 to 62.58)	2.24 (0.82 to 3.68)	0.002
NH White	15,886	44,664	181.15	53.73 (52.89 to 54.56)	110.94 (109.91 to 111.97)	3.21 (2.64 to 3.79)	< 0.001
NH American Indian	58	415	615.52	55.39 (41.84 to 71.93)	52.75 (47.62 to 57.89)	−0.84 (−2.15 to 0.48)	0.210
NH Asian or Pacific Islander	218	1421	551.83	34.44 (29.75 to 39.13)	50.84 (48.18 to 53.49)	1.17 (0.85 to 1.49)	< 0.001
Urbanization^1^							
Metropolitan	14,148	37,655	166.15	51.30 (50.46 to 52.15)	85.82 (84.95 to 86.69)	2.42 (2.12 to 2.73)	< 0.001
Nonmetropolitan	3,600	8,117	125.47	53.74 (51.98 to 55.50)	92.17 (90.16 to 94.19)	2.60 (2.08 to 3.12)	< 0.001
Age Groups^2^							
65-74 years	3,024	9,545	215.64	16.44 (15.85 to 17.02)	27.57 (27.02 to 28.13)	2.26 (1.77 to 2.75)	< 0.001
75-84 years	6,724	17,598	161.72	55.05 (53.73 to 56.36)	95.96 (94.54 to 97.38)	2.44 (1.81 to 3.07)	< 0.001
85+ years	8,000	25,126	214.08	192.70 (188.48 to 196.92)	406.05 (401.03 to 411.07)	2.94 (2.51 to 3.38)	< 0.001

“NH American Indian” denotes non-Hispanic American Indian or Alaska Native; “NH Asian or Pacific Islander” denotes non-Hispanic Asian or Pacific Islander.

^1^For urbanization, the 2023 AAMR was substituted with the 2020 value; AAPC was calculated for 1999–2020.

^2^For age groups, AAMR was replaced by the crude mortality rate, and AAPC was computed using the crude mortality rate.

AAMR, age-adjusted mortality rate; CI, confidence interval; AAPC, average annual percent change; NH, non-Hispanic.

Of the 52,269 total aggregate deaths in 2023, 79.1% (*n* = 41,341) were attributed to falls as the underlying cause of death, and 20.9% (*n* = 10,928) were attributed to MSDs (Table 2). Because underlying causes are coded mutually exclusively in the CDC WONDER system, there is 0% mathematical overlap of these codes on the UCOD level. This aggregate metric represents the combined total burden of these clinically related endpoints.

**Table 2:** Disaggregated underlying mortality burden and Joinpoint trend analysis for musculoskeletal disorders and falls (1999–2023).

Underlying cause of death	2023 deaths (%)	Trend segment 1 (APC)	*P*	Trend segment 2 (APC)	*P*	Trend segment 3 (APC)	*P*	Overall AAPC (95% CI)	*p*
**Falls only** (W00–W19)	41,341 (79.1%)	1999–2007: 6.09%	< 0.001	2007–2023: 3.19%	< 0.001	N/A	N/A	4.15% (3.72 to 4.58)	< 0.001
**MSDs only** (M00–M99)	10,928 (20.9%)	1999–2002: 2.07%	0.3128	2002–2017: −2.78%	< 0.001	2017–2023: 4.51%	< 0.001	−0.41% (−1.01 to 0.20)	0.192
**Combined composite**	52,269 (100.0%)	1999–2003: 5.21%	< 0.001	2003–2018: 1.75%	< 0.001	2018–2023: 4.41%	< 0.001	2.87% (2.34 to 3.40)	< 0.001

Deaths (%) represent the absolute death count and the relative proportion of each underlying cause within the composite endpoint for the terminal year 2023. Because underlying causes are assigned mutually exclusively in the CDC WONDER system, there is 0% mathematical overlap between Fall and MSD codes at the individual UCOD level.

APC, annual percent change; AAPC, average annual percent change; CI, confidence interval; MSDs, musculoskeletal disorders; UCOD, underlying cause of death.

**Figure 2:**
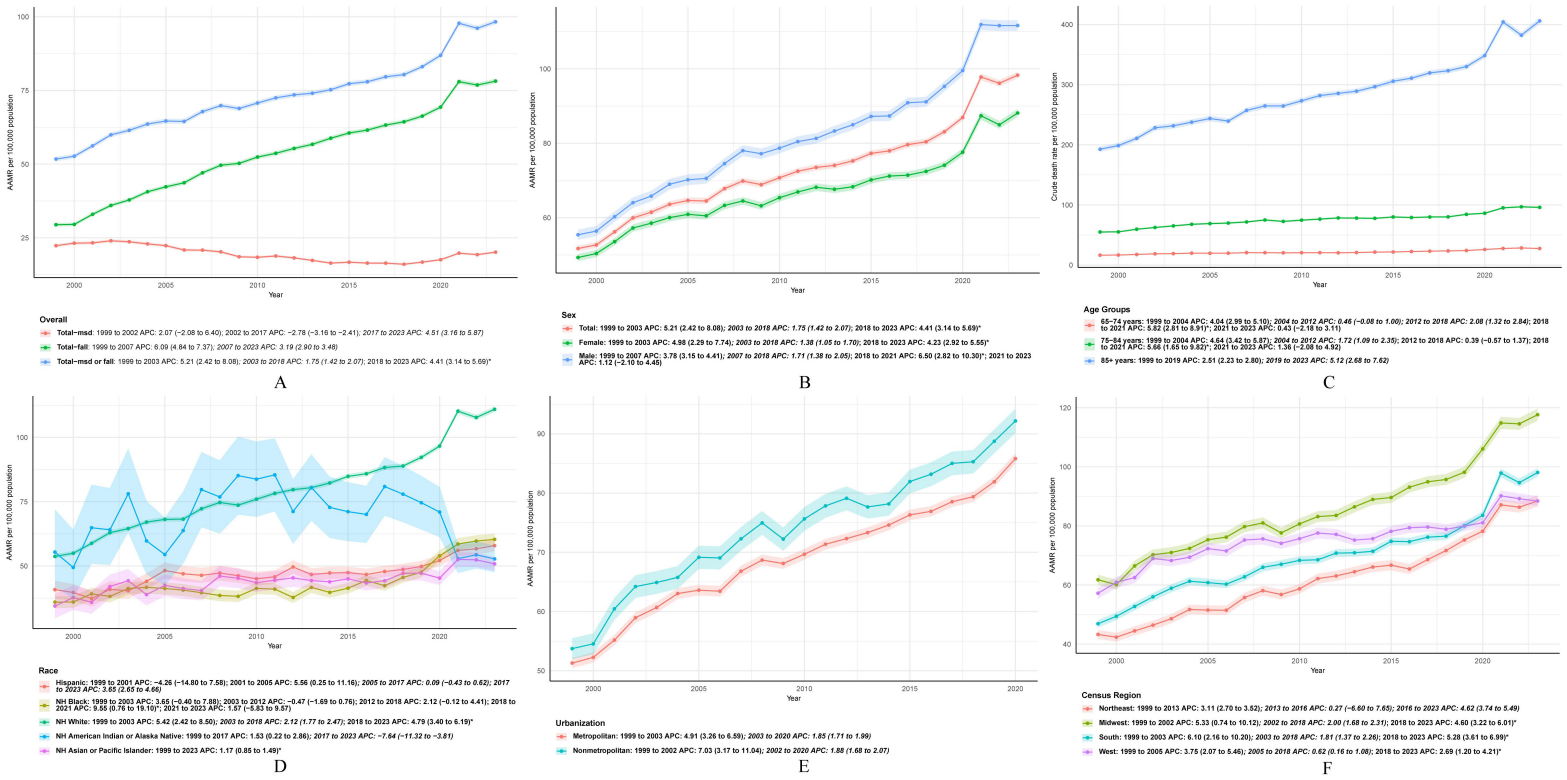
Temporal trends in mortality attributable to musculoskeletal disorders and falls among U.S. adults aged ≥65 years, 1999–2023. **(A)** Age-adjusted mortality rates (AAMRs) for musculoskeletal disorders (MSD) alone, falls alone, and the composite burden of MSD or falls. **(B–F)** Stratified temporal trends in the composite mortality burden (MSD or falls) by **(B)** sex, **(C)** age group (crude death rates), **(D)** race and ethnicity, **(E)** census region, and **(F)** urban-rural classification. Rates are per 100,000 population. Shaded bands represent 95% confidence intervals.

To understand the composite trend, we performed a disaggregated analysis (Table 2, Figure 2A). Fall-specific underlying mortality increased consistently throughout the study period (AAPC = 4.15%), with APCs of 6.09% (1999–2007) and 3.19% (2007–2023). Conversely, MSD-specific mortality exhibited a clinically highly relevant biphasic pattern: it declined significantly between 2002 and 2017 (APC = −2.78%, *p* < 0.001), but underwent a severe reversal thereafter, accelerating rapidly at an APC of 4.51% (*p* < 0.001) from 2017 to 2023.

### 3.2 Sex-specific trends

Joinpoint models identified phase-specific changes in the slope of mortality trends across different demographic categories (Figure 2B; Supplementary Table S1). For the total population, the composite mortality rate exhibited a sharp initial increase from 1999 to 2003 [APC = 5.21% (95% CI, 2.42 to 8.08), *p* < 0.001], followed by a moderate growth phase until 2018 [APC = 1.75% (95% CI, 1.42 to 2.07), *p* < 0.001], and culminating in a severe acceleration from 2018 to 2023 [APC = 4.41% (95% CI, 3.14 to 5.69), *p* < 0.001].

When stratified by sex, women demonstrated a consistently worsening trajectory, beginning with a significant rise between 1999 and 2003 [APC = 4.98% (95% CI, 2.29 to 7.74), *p* = 0.0010], slowing slightly from 2003 to 2018 (APC = 1.38%, *p* < 0.001), and surging again from 2018 to 2023 (APC = 4.23%, *p* < 0.001). Conversely, the trend among men experienced an initial increase from 1999 to 2007 (APC = 3.78%, *p* < 0.001) and a steady rise through 2018 (APC = 1.71%, *p* < 0.001). Notably, male mortality accelerated at a remarkable rate of 6.50% (95% CI, 2.82 to 10.30, *p* = 0.0018) between 2018 and 2021, before plateauing from 2021 to 2023 (APC = 1.12%, *p* = 0.4719). Although no formal statistical test of difference was conducted, the APC for men (6.50%) visibly exceeded that of women (4.23%) during the critical 2018–2021 period.

### 3.3 Age-specific trends

Mortality trajectories and absolute burdens differed substantially by age group (Figure 2C, Supplementary Table S1). The 65–74 age cohort demonstrated fluctuating trends, characterized by a significant early increase from 1999 to 2004 (APC = 4.04%, *p* < 0.001) and a stable period between 2004 and 2012 (APC = 0.46%, *p* = 0.0889). Rates for this group then rose steadily through 2018 (APC = 2.08%, *p* < 0.001), surged dramatically from 2018 to 2021 (APC = 5.82%, *p* = 0.0012), and stabilized again toward 2023 (APC = 0.43%, *p* = 0.728).

Similarly, older adults aged 75–84 experienced significant initial growth until 2012, followed by a non-significant plateau from 2012 to 2018 (APC = 0.39%, *p* = 0.388), and a subsequent sharp acceleration between 2018 and 2021 (APC = 5.66%, *p* = 0.010). In stark contrast, individuals aged ≥85 years faced a relentless and uninterrupted upward trajectory, with mortality rates growing steadily by 2.51% annually from 1999 to 2019 (*p* < 0.001), before steepening further to an APC of 5.12% from 2019 to 2023 (*p* < 0.001).

By 2023, clear age gradients were evident in the crude mortality rates, which escalated exponentially from 27.57 per 100,000 in the 65–74 age group, to 95.96 in the 75–84 group, and reaching an overwhelming 406.05 among those aged ≥85 years (Table 1). The corresponding average annual percent changes (AAPCs) over the entire 1999–2023 study period confirmed this age-dependent vulnerability, tracking at 2.26% (*p* < 0.001), 2.44% (*p* < 0.001), and 2.94% (*p* < 0.001) for the respective age tiers (Table 1).

### 3.4 Race/ethnicity

Temporal patterns varied significantly across racial and ethnic groups, highlighting persistent demographic disparities (Figure 2D, Supplementary Table S1). Among Hispanic older adults, the trajectory was characterized by a long period of stagnation from 2005 to 2017 (APC = 0.09%, *p* = 0.715), which was starkly reversed by a rapid and significant acceleration from 2017 to 2023 [APC = 3.65% (95% CI, 2.65 to 4.66), *p* < 0.001]. Non-Hispanic (NH) Black individuals experienced predominantly stable or slowly fluctuating rates for over a decade, until facing a dramatic surge between 2018 and 2021 [APC = 9.55% (95% CI, 0.76 to 19.10), *p* = 0.035].

Meanwhile, NH White adults showed a consistent and relentless pattern of worsening mortality, with rates increasing from 1999 to 2003 (APC = 5.42%, *p* = 0.0013), continuing through 2018 (APC = 2.12%, *p* < 0.001), and accelerating steeply from 2018 to 2023 (APC = 4.79%, *p* < 0.001). Interestingly, the NH American Indian/Alaska Native (AI/AN) population was the only group to demonstrate a recent significant decline, with rates dropping rapidly from 2017 to 2023 [APC = −7.64% (95% CI, −11.32 to −3.81), *p* < 0.001) following a prolonged period of moderate growth. Finally, the NH Asian/Pacific Islander (API) population maintained a steady, uninterrupted increase throughout the entire study period (AAPC = 1.17%, *p* < 0.001).

When assessing the population-adjusted burden, profound racial disparities were evident. In 2023, the absolute risk was highest among non-Hispanic (NH) White adults, who recorded an alarming AAMR of 110.94 per 100,000 population, followed by NH Black adults (60.34), and was lowest among NH AI/AN individuals (52.75). When evaluating the trajectory over the entire 1999–2023 study span, the NH White population also exhibited the most aggressive long-term worsening of population-adjusted risk (AAPC = 3.21%, *p* < 0.001) (Table 1). Conversely, the long-term AAPC for the NH AI/AN population remained statistically non-significant (−0.84%, *p* = 0.210), reflecting the protective effect of their recent sharp decline in mortality rates.

### 3.5 Urban–rural classification

When stratified by urbanization levels, metropolitan and non-metropolitan areas initially displayed distinct growth patterns that later converged (Figure 2E, Supplementary Table S1). Non-metropolitan areas experienced an aggressive early surge from 1999 to 2002 [APC = 7.03% (95% CI, 3.17 to 11.04), *p* = 0.0012]. Following this period, both metropolitan and non-metropolitan regions settled into nearly identical, sustained upward trajectories through 2020, with APCs of 1.85% (*p* < 0.001) and 1.88% (*p* < 0.001), respectively.

As previously detailed in the Methods, trend analyses for this specific geographic subgroup could not be extended to 2023. The CDC WONDER system systematically suppresses recent age-adjusted data for county-level urbanization queries to protect data stability. Crucially, while crude mortality rates were appropriate for narrow age-group analyses, we deliberately avoided substituting these missing AAMRs with crude rates for geographic trends. Urban and rural populations possess distinctly different and evolving age structures over the 25-year period; utilizing unadjusted crude rates would introduce severe age-structure confounding. Thus, terminating the rigorous, age-standardized analysis at 2020 remains the most epidemiologically sound approach.

### 3.6 U.S. census regions

Regional trajectories demonstrated considerable geographical heterogeneity across the United States (Figure 2F, Supplementary Table S1). The Northeast experienced a prolonged period of increase from 1999 to 2013 (APC = 3.11%, *p* < 0.001), followed by a brief stabilization, and a subsequent sharp rise from 2016 to 2023 (APC = 4.62%, *p* < 0.001). In the Midwest and South, early rapid growth phases were followed by steady increases through 2018; however, both regions experienced severe late-stage accelerations from 2018 to 2023, reaching APCs of 4.60% (*p* < 0.001) and 5.28% (*p* < 0.001), respectively. The West followed a comparatively milder trajectory, though it still concluded with a significant increase from 2018 to 2023 (APC = 2.69%, *p* = 0.011).

When standardizing for the significant population growth and demographic shifts across different U.S. regions, the South still emerged as the epicenter of this escalating crisis (Table 1). By 2023, the highest AAMR was recorded in the Midwest (117.65 per 100,000) and the South (98.08), while the Northeast (88.47) and West (88.41) reported similar, lower rates. However, when evaluating the velocity of deterioration over the entire 1999–2023 period, the South experienced the most aggressive long-term growth in population-adjusted mortality risk (AAPC = 3.23%, *p* < 0.001), significantly outpacing the West, which recorded the lowest overall growth rate (AAPC = 1.82%, *p* < 0.001) (Table 1).

### 3.7 State-level differences

**Figure 3:**
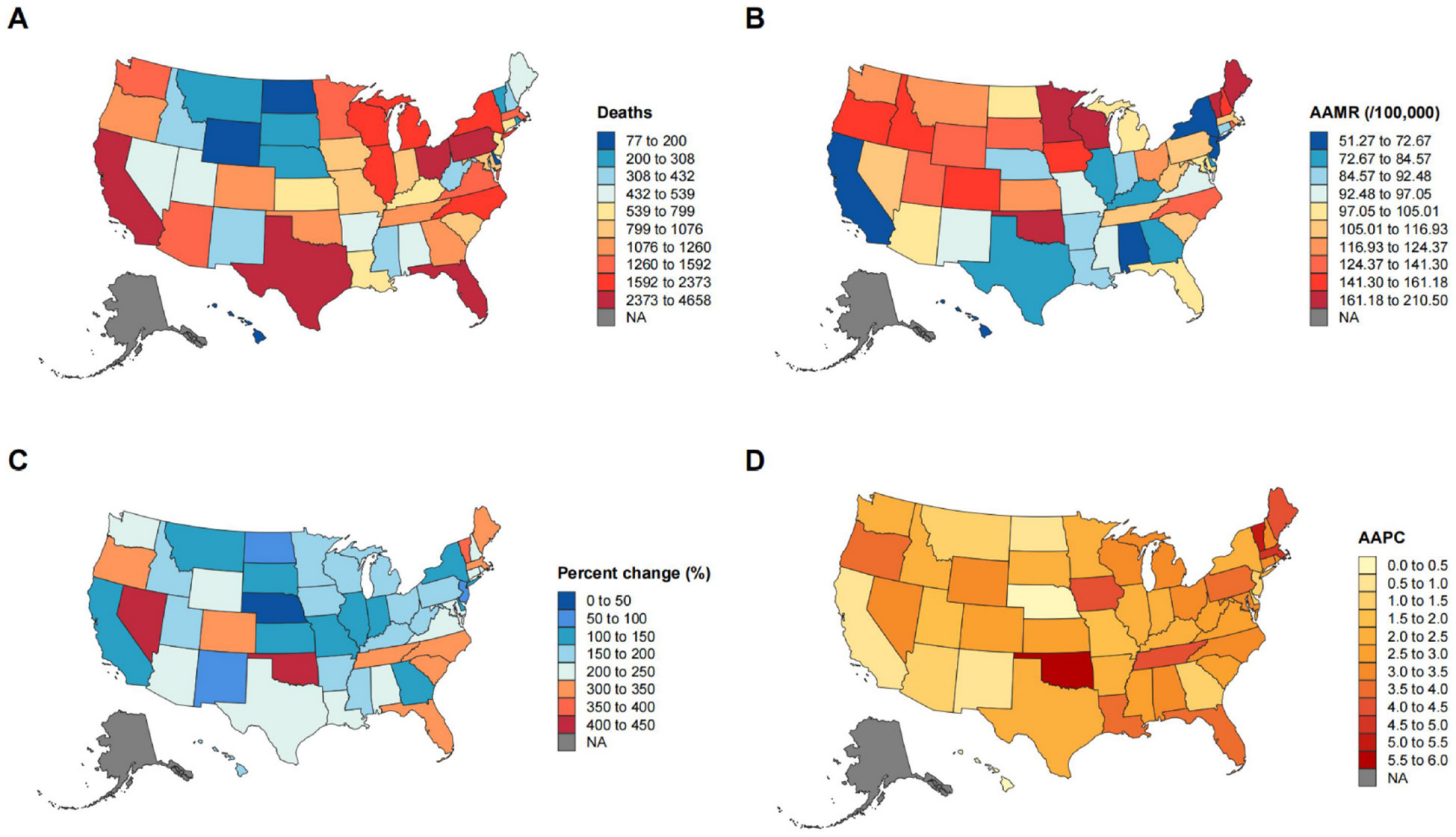
Geographic variations in deaths, mortality rates, and trends of musculoskeletal diseases and falls among US older adults by state. **(A)** State-level distribution of deaths, **(B)** age-adjusted mortality rates, **(C)** percentage changes in death counts, **(D)** average annual percentage changes.

At the state level, the true epidemiological burden and historical trajectories varied widely across two critical risk-standardized metrics: the 2023 AAMR and the long-term AAPC (Figure 3, Supplementary Table S2).

The 2023 AAMR revealed a stark 3.3-fold disparity between the highest-burden state and the lowest. Oklahoma recorded an overwhelming AAMR of 184.94 per 100,000, while California reported the lowest rate at 55.59. Notably, some states entered the study period with an already high baseline risk (e.g., Idaho, AAMR 100.23 in 1999), whereas others experienced a rapid accumulation of burden, such as Connecticut, where the age-adjusted rate more than doubled from 37.77 in 1999 to 87.28 in 2023.

Regarding the velocity of this worsening burden, several states significantly outpaced the national AAPC estimate of 2.87%. Oklahoma (5.70%), Vermont (5.30%), and Tennessee (4.31%) recorded the steepest historical trajectories, all of which did not overlap with the national 95% CI and were statistically significant (*p* < 0.05). Conversely, Nebraska (0.26%, *p* = 0.299), Hawaii (0.41%, *p* = 0.133), and New Mexico (0.64%, *p* = 0.625) reported the lowest long-term growth rates in the nation.

## 4 Discussion

The present study analyzed a comprehensive national mortality attributed jointly to musculoskeletal disorders and falls among U.S. adults aged 65 years and older, revealing a sustained long-term increase with marked heterogeneity by age, sex, race/ethnicity, geography, and urbanicity. Extending surveillance through 2023 and applying segmented log-linear models, we identify periods of deceleration and renewed acceleration that would be obscured by single-slope summaries. The overall rise in age-adjusted mortality aligns with the demographic reality of population aging but exceeds what would be expected from demographic change alone, indicating a dynamic interplay between physiological vulnerability, multimorbidity, clinical practice patterns, and environmental exposures (7). Importantly, the inflection that began in the late 2010s was evident across multiple strata, underscoring that the recent upswing was not limited to a single subgroup and that prevention strategies anchored solely in historical baselines may underestimate contemporary risk (7).

The most profound clinical shift uncovered in this study emerges from our disaggregated analysis (Table 2). The historical decline in MSD-specific mortality (APC = −2.78% from 2002 to 2017) likely reflects major therapeutic advancements during that era, particularly the widespread adoption of bisphosphonates for osteoporosis and targeted biologics for rheumatologic diseases (30). However, this success was sharply reversed post-2017, transitioning into a concurrent crisis where both MSD and fall mortalities are currently surging at alarming rates (APCs of 4.51% and 3.19%, respectively). Furthermore, tracking the composite burden is conceptually essential even during the historical period (2002–2017) when the specific underlying trajectories diverged (MSD mortality declining while fall mortality rose). One might argue that aggregating these diverging trends masks the isolated clinical success of pharmacological MSD management during that era. However, from a macro-level health system perspective, evaluating them together reveals a more profound truth: the therapeutic gains in slowing intrinsic bone disease (MSDs) were entirely eclipsed by the surging lethality of external biomechanical triggers (falls) (31, 32). Moreover, this aggregate metric prevents 'diagnostic shifting' from obscuring the true public health burden. While pharmacological interventions may have improved bone density enough to prevent spontaneous MSD-coded deaths, older adults' skeletal reserves were still insufficient to withstand traumatic impacts, resulting in fatal falls (33). By conceptualizing MSDs and falls as a unified “musculoskeletal and mobility failure” endpoint, our findings demonstrate that the ultimate downstream demand on orthopedic and geriatric resources never actually declined, highlighting the persistent inadequacy of single-pathway interventions. Crucially, the inflection point for this synergistic surge (2017) definitively predates the COVID-19 pandemic, which was first identified in late December 2019 and declared a global pandemic on March 11, 2020. This chronological sequence is vital: it demonstrates that the concurrent acceleration is not merely an anomalous artifact of the pandemic, but rather evidence of a systemic breakdown that had already begun under normal pre-pandemic conditions. It indicates that the piecemeal, fragmented care models of the past—treating bone density in the clinic while addressing fall hazards in the community—had already reached their limits by the late 2010s. Because this structural failure predates the viral outbreak, it highlights exactly why isolated surveillance is no longer sufficient. Monitoring these endpoints in silos masks the compounding nature of a crisis that was already brewing. When the pandemic did arrive, it acted as a severe, targeted catalyst that exponentially worsened this pre-existing vulnerability. Lockdowns, social distancing mandates, and deferred routine orthopedic care resulted in prolonged immobility, which rapidly accelerated sarcopenia, frailty, and bone mineral loss in older adults (34, 35). Consequently, a unified metric is essential to quantify the true, overwhelming demand placed on downstream healthcare resources, including orthopedic surgery and geriatric rehabilitation. We acknowledge that the rising incidence of falls is undoubtedly multifactorial—driven by population-level trends in obesity, polypharmacy, and neurocognitive decline. However, the lethality of these falls remains inextricably linked to the patient's underlying musculoskeletal reserve (36). Regardless of whether a fall is proximally triggered by a mechanical hazard, a medication side-effect, or altered biomechanics from increased body mass, the ultimate mortality outcome reflects a systemic failure of skeletal resilience and muscle strength to withstand the traumatic impact (37). As conceptualized in our “disease–injury–disability” framework (Figure 1), both underlying causes ultimately converge on the exact same endpoint: catastrophic loss of mobility. Therefore, tracking their aggregate underlying mortality provides a methodologically valid and practically essential macro-level metric of this end-stage vulnerability.

The age pattern of this mortality burden is biologically expected yet policy-critical. Adults aged 85 years and older consistently experienced the highest absolute rates. However, the finding that the youngest older group (65–74 years) also exhibited a transient mortality surge during 2018–2021 indicates that modifiable risk accumulates earlier than many clinical programs assume. Initiating structured strength, balance, and bone-health interventions at the first evidence of functional decline—rather than waiting until the mid-to-late 70s—may yield substantial absolute survival gains (34, 38). Sex differences, particularly the sharper increase among men from 2018 to 2021, are compatible with interactions between physiological trajectories (e.g., rapid loss of muscle power) and distinct health-seeking behaviors (7). Conversely, because women constitute a larger share of the oldest-old, their cumulative lifetime exposure to the highest-risk age bands is greater, reinforcing the need to time interventions alongside menopause-related changes in body composition and bone health (39). Racial, ethnic, and geographic patterns further illustrate this stark heterogeneity. The short-term acceleration among non-Hispanic Black populations during 2018–2021 likely reflects a convergence of structural determinants, including higher chronic disease burdens, barriers to continuous primary care, and limited local post-acute rehabilitation resources. Geographically, terminal AAMRs remain highly concentrated in the Midwest, while states in the South exhibit AAPCs exceeding the national average, signposting locales where trajectories are steepening rapidly. Furthermore, our urban–rural analysis (1999–2020) demonstrated a sharper early-period increase in non-metropolitan areas, underscoring that infrastructure deficits and emergency response constraints remain binding limitations that clinical interventions alone cannot fully offset (40).

Beyond epidemiological characterization, these results dictate practical shifts in intervention design. First, surveillance must routinely employ segmented time-series methods to prevent miscalibrating program intensity when historical trajectories change abruptly (28). Second, prevention windows must shift earlier, utilizing multicomponent programs that combine progressive resistance training with hazard remediation and deprescribing at or shortly after age 65 (34, 39). Third, implementation must be equity-oriented, directing targeted investments in bone density testing and post-fall rehabilitation toward the most affected demographics and regions to mitigate widening disparities.

The strengths of this study include its 25-year horizon capturing multiple policy eras, harmonized age adjustment across strata, and granular multimetric state-level presentation that explicitly quantifies statistical uncertainty. However, several limitations require careful consideration to inform future research. First, our analysis relied exclusively on the Underlying Cause of Death (UCOD) rather than Multiple Cause of Death (MCOD) records. This was a deliberate methodological choice designed to capture the initiating event of the fatal cascade (dying from an MSD or fall) and to avoid the massive co-morbidity bias that occurs when ubiquitous background conditions (e.g., incidental osteoarthritis) are recorded alongside unrelated fatal events like acute myocardial infarction. Furthermore, the mutually exclusive nature of UCOD coding provided a mathematically clean foundation for our composite endpoint, ensuring zero double-counting. However, as a trade-off, we could not evaluate the exact proportion of deaths where MSDs and falls co-occurred on the identical death certificate (e.g., an MSD as a contributing cause to a fatal fall). While our composite endpoint successfully captures the aggregate macro-level burden, future studies utilizing MCOD microdata are warranted to quantify individual-level pathological overlap. Second, our methodological decision to exclude Alaska—necessitated by unstable, sub-20 annual death counts that generated statistically unreliable AAMRs—introduces a specific demographic limitation. Because Alaska is home to a substantial proportion of the U.S. Native population, this exclusion systematically biases the estimates for the non-Hispanic American Indian/Alaska Native (NH AI/AN) subgroup. The absolute mortality burden for the NH AI/AN population reported here is likely an underestimation, and temporal trends for this demographic should be interpreted with caution. Third, due to system-level restrictions in the database that disable automated age-adjusted rate calculations for recent county-level queries, our urban-rural stratification was truncated at 2020. Consequently, while we captured the initial onset of the late-2010s acceleration, we cannot definitively evaluate how the urban-rural disparity evolved during the later stages of the pandemic (2021–2023). Future validation using unsuppressed microdata is required. Finally, standard age adjustment does not fully account for compositional shifts within the ≥85 group, and the ecological design prevents inference regarding individual-level mechanisms. This points directly to a constructive research agenda: linkage of mortality records with Medicare claims, pharmacy fills, and rehabilitation encounters will enable the decomposition of mortality trends into proximal risk pathways (e.g., bone-protective therapy gaps, sedative exposure) (39, 41).

## 5 Conclusion

Mortality at the intersection of musculoskeletal disorders and falls among older Americans has risen meaningfully over the past quarter-century, culminating in a severe, concurrent acceleration since 2017. By conceptualizing this as a unified endpoint, this study demonstrates that isolated clinical management—such as pharmacological bone fortification without concurrent mobility preservation—is fundamentally insufficient. These findings demand an urgent paradigm shift toward integrated orthopedic-geriatric co-management models (e.g., Fracture Liaison Services). Clinically, this requires mandated bidirectional screening: older adults treated for chronic MSDs must undergo comprehensive fall-risk assessments (including polypharmacy review and sarcopenia evaluation), while fall victims necessitate immediate bone health optimization. At the policy level, the disproportionate burden concentrated in the South and non-metropolitan areas dictates geographically targeted resource allocation. Policymakers should restructure Medicare and Medicaid reimbursement frameworks to fund not only post-acute surgical rehabilitation, but also proactive home environment modifications and community-based strength-and-balance programs. Ultimately, mitigating this escalating “disease-injury-disability” cycle requires a synchronized, multimodal public health effort to preserve both the structural resilience and physical independence of an aging society.

## Data Availability

The raw data supporting the conclusions of this article will be made available by the authors, without undue reservation.
